# Parkinson Disease Pathogenic Variants: Cross-Ancestry Analysis and Microarray Data Validation

**DOI:** 10.1212/NXG.0000000000200379

**Published:** 2026-04-13

**Authors:** Samantha Hong, Mathew J. Koretsky, Jens Lichtenberg, Hampton L. Leonard, Vanessa Pitz, as the Global Parkinson's Genetics Program (GP2)

**Affiliations:** 1 Center for Alzheimer's and Related Dementias, NIH, Bethesda, MD;; 2 DataTecnica LLC, Washington DC;; 3 Molecular Neurogenetics Section, Medical Genetics Branch, National Human Genome Research Institute, NIH, Bethesda, MD; and; 4 Integrative Neurogenomics Unit (INU), Laboratory of Neurogenetics, NIH, Bethesda, MD.

## Abstract

**Background and Objectives:**

Known pathogenic variants (PVs) in Parkinson disease (PD) contribute to disease development but have yet to be fully explored by arrays on a large scale. This study evaluated genotyping success of the NeuroBooster array (NBA) and determined the frequencies of PVs across ancestries.

**Methods:**

We analyzed the presence and allele frequency of PVs in 28,710 PD cases, 9,614 other neurodegenerative disorder cases, and 15,821 controls across 11 ancestries within the Global Parkinson's Genetics Program (GP2) data set. Cluster plots were used to assess the quality of PVs genotyped on NBA.

**Results:**

Genes previously predicted to have high or very high confidence of causing PD tend to have more PVs and are present across ancestry groups. Of 34 known PD gene PVs assessed, 25 were typed by NBA and classified as “good” (n = 12), “medium” (n = 4), or “bad” (n = 9) quality variants.

**Discussion:**

Our results confirm the likelihood that established PD genes are pathogenic and highlight the importance of ancestrally diverse research in PD. We also show the usefulness of the NBA as a reliable tool for the genotyping of rare variants of PD.

## Introduction

With the expanding global burden of Parkinson disease (PD), investigating causes, risk factors, and potential therapeutic targets becomes increasingly important. Most cases of PD occur sporadically, arising from interplay of genetic and environmental factors.^1-3^ Pathogenic variants (PVs), both common and rare, often cause functional changes in critical proteins, increasing the risk of developing disease. In PD, PVs have been found in well-known genes such as *LRRK2*, *PRKN*, *PINK1*, and *SNCA*, which have been linked to familial and sporadic forms of the disease.^4-7^

Recent developments in next-generation sequencing techniques have facilitated the discovery of PVs. A recent PD genome-wide association study meta-analysis of European individuals uncovered 90 risk loci.^8^ Efforts have been made to conduct genetic studies of PD in non-European populations, such as in East Asian, Latino, and African ancestries. These populations have been underrepresented in genetics research because of historical exclusion and barriers to study recruitment and access. Expanding knowledge of PD's genetic influence in underrepresented ancestries is crucial for understanding the disease.^9-11^

A recent review categorized 21 genes with reported PVs according to their likelihood of causing PD^12^. This review, along with much of the research in PD genetics, has been primarily focused on samples of European ancestry.^13^ The NeuroBooster array (NBA) is a custom platform aimed to genotype variants of neurologic conditions across diverse populations.^14^ In this study, we evaluate the success of NBA and analyze frequencies of PVs in diverse populations leveraging data from the Global Parkinson's Genetics Program (GP2).

## Methods

### The Global Parkinson's Genetics Program

Data for this study were obtained from GP2, which is a global initiative focused on improving the understanding of the genetic architecture of PD through the collection of ancestrally diverse samples. We used samples in GP2 release 7 to investigate PVs that have been studied in predominantly European populations across a larger set of ancestries. The data consisted of 28,710 PD cases, 9,614 other neurodegenerative disorder (NDD) cases, and 15,821 controls across 11 ancestry groups: African American (AAC), African (AFR), Ashkenazi Jewish (AJ), Admixed American/Latin American (AMR), Complex Admixture History (CAH), Central Asian (CAS), East Asian (EAS), European (EUR), Finnish (FIN), Middle Eastern (MDE), and South Asian (SAS) (eTable 1).^15^ All GP2 samples were genotyped on NBA, which was developed from a backbone of 1.9 million variants and includes custom content consisting of 95,000+ variants associated with more than 70 neurologic conditions and 10,000 tagging variants to facilitate imputation across diverse populations.^14^

### Data Processing

**Figure 1:**
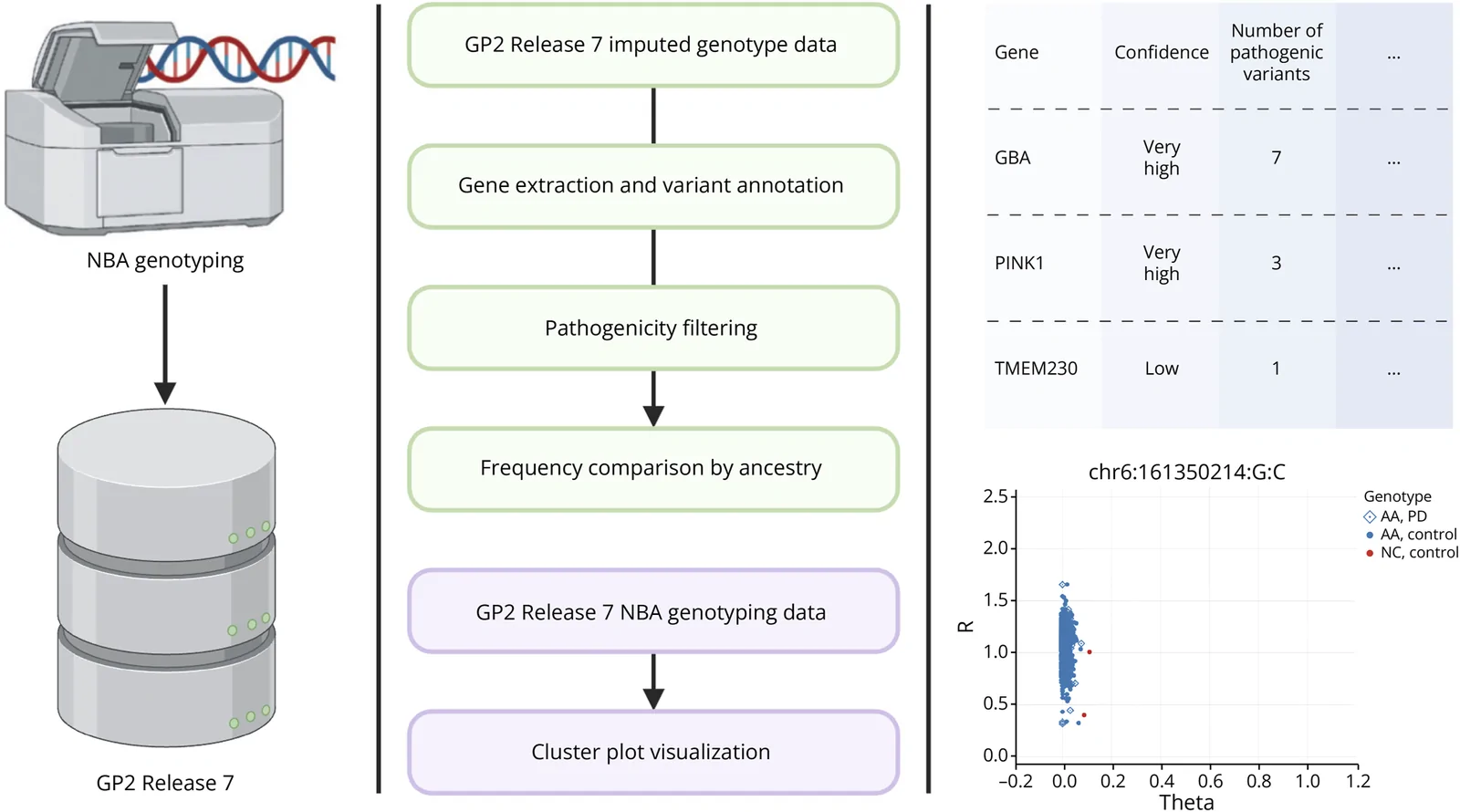
Workflow Overview GP2 = Global Parkinson's Genetics Program, GWAS = genome-wide association study.

We investigated variants in 21 PD genes: *SNCA, PRKN, UCHL1, PARK7, LRRK2, PINK1, POLG, HTRA2, ATP13A2, FBX07, GIGYF2, GBA1, PLA2G6, EIF4G1, VPS35, DNAJC6, SYNJ1, DNAJC13, TMEM230, VPS13C, *and* LRP10*.^12^ All variants in the GP2 imputed data were annotated across each ancestry using ANNOVAR (version 2020-06-07) and ClinVar (version 2024-09-17). Variants labeled “pathogenic” in the “clinical significance” field within the genes of interest were extracted using PLINK1.9 (eTable 2).^16,17^ To ensure a conservative definition of PVs and minimize heterogeneity in downstream analyses, we restricted our analysis to ClinVar entries labeled strictly as “pathogenic”, excluding those annotated as “pathogenic/likely pathogenic” or “likely pathogenic”. PLINK was also used to calculate the frequencies of the PVs across ancestries. For context, we compared the PVs identified in our data set with allele frequencies from gnomAD (v4.1.0), using this resource as a population-level reference across ancestries rather than for disease-specific interpretation. We produced cluster plots to assess genotyping quality of NBA for the PVs of interest on the GP2 samples. Cluster plots visualize the different allelic statuses (homozygous reference/alternate, heterozygous) by plotting Theta and R values that are provided by Illumina (GenomeStudio v2.0.5) to visually assess the genotyping accuracy.^18,19^ We used 2 criteria to assess genotype success of a variant based on clustering quality: (1) shape and distinction of the clusters and (2) number of samples for which the variant was not called (NCs). A plot was labeled as “bad” if it did not follow the normal “spread” in regard to cluster distance (change in Theta between clusters ≤ 0) or if many NCs (low call rate) were observed (NCs ≥ 10). If a plot exhibited expected behavior, it was classified as “good”. Plots that were difficult to classify or where analysts' responses differed were labeled as “medium”. Although several software tools exist for automatic genotype clustering, they have been shown to perform inconsistently for rare variant calls. Manual scoring using the predefined criteria for cluster separation and call rate therefore provided a more reliable evaluation approach in this context. Figure 1 presents an overview of the workflow.

### Standard Protocol Approvals, Registrations, and Patient Consents

This study did not involve the recruitment of human participants or the use of animal subjects. All analyses were conducted on deidentified data obtained from GP2 and from the Accelerating Medicine Partnership PD (AMP PD), which is available to qualified researchers on application (see Data Availability for details). Data collection for GP2 was approved by the relevant institutional ethics committees and by the original investigators. Our use of the data complied with all applicable terms of access and use.

### Data Availability

Data used for this article were obtained from GP2 and from the AMP PD Knowledge Platform (DOI 10.5281/zenodo.10962119, release 7). A repository containing all code for data processing and analysis is publicly available to facilitate replication (DOI 10.5281/zenodo.14193209).

## Results

A total of 34 labeled PVs from the 21 genes of interest were analyzed (eTable 2). Only 10 of the PVs within the established PD genes had some form of PD denoted within the “clinical disease name” field on ClinVar. The other 24 PVs were clinically associated with other NDDs or labeled as “not provided.”

To assess the genotyping quality of PVs using NBA, we created cluster plots for each nonimputed variant and had 2 analysts visually inspect them. A total of 25 of the 34 PVs were typed (nonimputed) and thus visualized through a cluster plot. Examples of cluster plots that were classified as “good,” “medium,” and “bad” can be found in eFigure 1.

After classification, there were 12 variants labeled as “good,” 4 labeled as “medium,” and 9 labeled as “bad” (eTable 3). The number of NCs for each typed variant was also included, along with the minor allele frequency and missingness rate for that variant in GP2. Images of all 25 cluster plots can be found at: DOI 10.5281/zenodo.14193209.

Table 1 contains the distribution of the identified PVs across the genes of interest. There are 4 genes that were classified as having a “very high” confidence for causing PD, *FBXO7*, *SNCA*, *PARK7*, and *VPS35*, which had zero PVs.^12^ Six of the 7 genes that were classified as “low” confidence for causing PD also had zero PVs present across all ancestries; *TMEM230* was the only “low” probability gene that had a PV present. To further assess NBA's ability to capture PVs; we compared the variants identified within each gene with those reported in gnomAD as a reference for allele frequencies across ancestries (eTable 4). It is important to note that this comparison is not intended to imply clinical or disease-specific relevance.

**Table 1:** Number of Pathogenic Variants by Gene and Ancestry, With Gene-Level Confidence of PD Associations^12^

Confidence as actual PD gene per Blauwendraat et al. 2020	Gene	Total number of NBA pathogenic variants	AAC	AFR	AJ	AMR	CAH	CAS	EAS	EUR	FIN	MDE	SAS
Very high	SNCA	0	0	0	0	0	0	0	0	0	0	0	0
Very high	PRKN	5	0	0	1	1	1	0	1	1	0	0	0
Very high	PARK7	0	0	0	0	0	0	0	0	0	0	0	0
Very high	LRRK2	1	0	0	0	0	0	1	0	1	0	1	0
Very high	PINK1	3	0	0	0	0	0	0	2	2	1	1	0
Very high	ATP13A2	5	0	1	0	1	0	0	1	3	0	0	0
Very high	FBXO7	0	0	0	0	0	0	0	0	0	0	0	0
Very high	GBA1	7	0	2	0	0	1	0	0	4	0	1	0
Very high	PLA2G6	3	0	0	0	1	0	1	1	1	0	0	0
Very high	VPS35	0	0	0	0	0	0	0	0	0	0	0	0
High	POLG	8	2	4	1	0	0	0	0	6	0	0	0
High	DNAJC6	0	0	0	0	0	0	0	0	0	0	0	0
High	SYNJ1	1	0	1	0	0	0	0	0	0	0	0	0
High	VPS13C	0	0	0	0	0	0	0	0	0	0	0	0
Low	UCHL1	0	0	0	0	0	0	0	0	0	0	0	0
Low	HTRA2	0	0	0	0	0	0	0	0	0	0	0	0
Low	GIGYF2	0	0	0	0	0	0	0	0	0	0	0	0
Low	EIF4G1	0	0	0	0	0	0	0	0	0	0	0	0
Low	DNAJC13	0	0	0	0	0	0	0	0	0	0	0	0
Low	TMEM230	1	0	0	0	0	0	0	0	1	0	0	0
Low	LRP10	0	0	0	0	0		0	0	0	0	0	0

Abbreviations: AAC = African American; AFR = African; AJ = Ashkenazi jewish; AMR = Admixed American/Latin American; CAS = Central Asian; EAS = East Asian; EUR = European; FIN = Finnish; MDE = Middle Eastern; SAS = South Asian.

A breakdown of all PVs identified by NBA across the different ancestries shows the number of variants unique to a specific ancestry is biased toward the EUR population, which has 11 unique variants (eTable 5). Of further relevance is that the SAS ancestry group had no PVs present within the PD genes of interest. Also of interest, each of the PVs within *PRKN* were found to be present in only 1 population.

## Discussion

Analyzing the genetic influence of PVs across diverse populations is important to enhance our understanding of PD.^20^ In this study, we evaluated the effectiveness of NBA for genotyping PVs through cluster plot and allele frequency analyses.

The cluster plot analysis revealed that NBA is an effective platform for studying PD across diverse populations. The number of NCs for PVs was high in genes that are historically difficult to genotype. High-quality genotyping is vital for accurately identifying PVs, yet it is often assumed to be adequate. For example, the pathogenic gene *GBA1* is located in a region with a complex sequence, many copy number variants, and the nearby pseudogene *GBAP1*, which shares high sequence similarity with *GBA1* and contributes to misalignment and errors during genotyping.^21^ Although NBA is an advanced tool for genotype analysis for NDDs, the existence of “medium” and “bad” variants underscores the importance in verifying genotype quality.

We found more PVs in genes classified as having a “very high” probability of causing PD compared with genes with “low” probability. Many of the genes that were classified as having “low” confidence of causing PD had no PVs present in our data. It must be noted that NBA was designed to capture PVs in established PD genes, which could contribute to more PVs being found in genes with a “very high” influence on PD risk. Of interest *SNCA*, *FBXO7*, *VPS35*, and *PARK7* were found to have no PVs present in our data despite their “very high” confidence in contributing to PD. It is possible that our understanding of the pathogenicity of these variants has changed over time and that ClinVar annotations should be updated to reflect this. In addition, some variants may simply be extremely rare and therefore difficult to genotype or capture in available data sets.

It is noteworthy that certain PVs are present only in specific ancestries. For example, the single annotated PV in *LRRK2* (rs33939927) is present in only the CAS, EUR, and MDE populations. The single annotated PV in *TMEM230*, a gene classified as having “low” confidence in contributing to PD, is only present in EUR population. This may be because many genetic studies identifying pathogenic genes for PD have historically focused on samples of European ancestry, skewing the importance of these genes and not representing their global significance. A low frequency of variants in ancestries other than EUR may also exist because the diverse ancestry sample size is notably smaller than the EUR ancestry in GP2. It is also interesting to note cases where PVs of a gene are present in unique ancestries, such as in *GBA1*. This underscores the importance in conducting research on ancestrally diverse samples, corroborating previous claims of the lack of population diversity in PD research.^13^

Some limitations of our study include the fact that some PVs were imputed using the TOPMed Imputation Server instead of being directly typed from NBA.^22^ We were therefore unable to create cluster plots for these PVs. In addition, the NBA cluster plot categorization was performed through manual evaluation, which may introduce bias in the classification of “medium” variants.

Although our study included a variety of ancestral backgrounds to combat the lack of diversity in the study of PD genes, our largest demographic was still EUR with 38,839 samples. Our reliance on ClinVar to denote PVs limits our analysis to variants within the scope of the ClinVar annotations. ClinVar may be biased as it labels PVs from studies primarily published in European populations. Some future directions that may come from this work include studies focusing on the monogenic effect of genes with PVs that are unique to specific ancestries, which will be important for ancestry-informed risk assessment.

GP2's initiative to provide more comprehensive, diverse populations for studying PD has clinical and practical implications in the study of PD genetics. Our study reveals that conventional PD genes that were identified in predominantly European populations may not have the same implications in non-European ancestries. In addition, our evaluation of NBA's genotyping success underscores its effectiveness in identifying these variants. As a screening tool, NBA offers important strengths including scalability and the ability to capture genetic variation across diverse ancestries at a comparatively low cost. At the same time, NBA has inherent limitations, particularly reduced accuracy in regions with higher sequence complexity, gaps in coverage for certain variant types, and reliance on existing variant annotations. While sequencing approaches remain the gold standard for comprehensive variant detection, NBA provides an efficient and scalable platform for initial population-level screening, complimenting deeper sequencing efforts. This work highlights the critical role of GP2 in enhancing our understanding of PD genetics across diverse populations and underscores NBA's utility in robustly detecting PVs.

## Glossary

AMP PD: accelerating medicine partnership PD
CAS: Central Asian
GP2: genetics program
MDE: middle eastern
NBA: NeuroBooster array
NC: not called
NDD: neurodegenerative disorder
PD: Parkinson disease
PV: pathogenic variant
SAS: South Asian
